# Genome‐Wide In Silico Analysis of the Type VI Secretion System (T6SS) Within the *Morganella* Genus

**DOI:** 10.1002/mbo3.70304

**Published:** 2026-04-30

**Authors:** Mathilde Duque, Aymeric Jacquemin, Delphine Girlich, Rémy A. Bonnin, Laurent Dortet

**Affiliations:** ^1^ INSERM, CEA, Center for Immunology of Viral, Auto‐Immune, Hematological and Bacterial Diseases » (IMVA‐HB/IDMIT/UMRS1184) Paris‐Saclay University Fontenay‐aux‐Roses & Le Kremlin‐Bicêtre France; ^2^ Bacteriology‐Hygiene Unit, Bicêtre Hospital Assistance Publique‐Hôpitaux de Paris Le Kremlin‐Bicêtre France; ^3^ SEPSIS Comprehensive Center, IHU SEPSIS; ^4^ Associated French National Reference Center for Antibiotic Resistance: Carbapenemase‐Producing Enterobacteriaceae Le Kremlin‐Bicêtre France

**Keywords:** host–pathogen interaction, in silico characterization, *Morganella* spp., type VI secretion system (T6SS)

## Abstract

*Morganella* spp., part of the *Morganellaceae* family, are opportunistic pathogens commonly found in the human gut microbiota. They are highly adaptable, cause various types of infections, and are difficult to treat due to virulence factors and resistance genes. In 2017, *Morganella* became part of the WHO Bacterial Pathogen Priority List (BPPL). In 2024, a genomic study revealed significant differences between clinically relevant species, particularly between *M. sibonii* and *M. morganii*, with *M. sibonii* possessing a unique type VI secretion system (T6SS) operon. This study aimed to explore the distribution and diversity of T6SS in *Morganella* species. A retrospective analysis of 293 *Morganella* genomes from 1966 to 2023 was conducted. The T6SS clusters and subtypes were annotated using the SecReT6 platform and RAST, with phylogenetic analysis performed on *tssB* genes. Putative effectors and immunity proteins were identified through in silico analysis using BlastP and Alphafold. We identified that *M. sibonii* isolates possess four T6SS clusters of subtypes i1, i3 v.1, i3 v.2, and i5. In contrast, most *M. morganii* isolates lacked T6SS, with only one‐third containing a subtype i1 T6SS. Notably, *M. morganii* frequently (95%, 117/123 effectors) harbored a T6SS‐predicted TseV effector, while *M. sibonii* exhibited a greater diversity of effectors. This study highlights species‐specific T6SS patterns among *Morganella* genus and suggests that T6SS might play a crucial role in bacterial lifestyle competition and host–pathogen interactions. It paves the way for future research on *Morganella* pathogenicity.

## Graphical abstract

▪ Species‐specific T6SS patterns among *Morganella* genus (*M. morganii* and *M. sibonii*).
▪ Only one‐third of *M. morganii* isolates haboured a T6SS.
▪ All *M. sibonii* possess four T6SS clusters.
▪ *M. sibonii* exhibited a wide diversity of effectors compared to *M. morganii.*

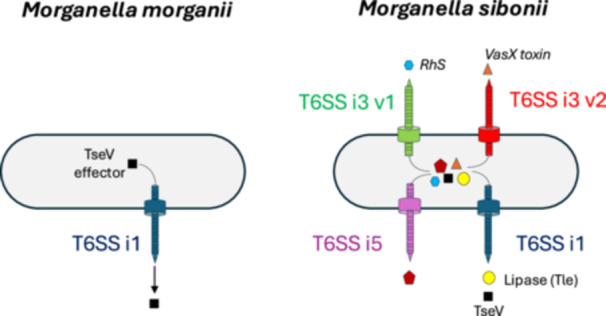

## 1 Introduction

### 1.1 *Morganella*: An Increasingly Recognized Pathogen

*Morganella* isolates are motile, facultative anaerobic, Gram‐negative rods (Morgan 1906), widely distributed in the environment and are part of the gut microbiota of humans, mammals, and reptiles (Lee et al. 2009). It is an opportunistic pathogen, and numerous reports have documented its implications in various human pathologies such as sepsis, abscesses, purple urine bag syndrome, chorioamnionitis, and cellulitis (Bandy 2020). This bacterium naturally produces a cephalosporinase named DHA, which confers resistance to ampicillin, amoxicillin, and most first‐ and second‐generation cephalosporins. Additionally, *Morganella* spp. exhibits low susceptibility to imipenem due to the low affinity of its penicillin‐binding protein, PBP‐2 (Bonnin et al. 2021).

Furthermore, since 2017 “*Morganella* spp. resistant to third‐generation cephalosporin” has been part of the World Health Organization Bacterial Pathogen Priority List (WHO BPPL), and it has been categorized as critical in the WHO BPPL of 2024. Its incidence is under 100 cases/million population, and its mortality is about 5%–10% (WHO Bacterial Priority Pathogens List 2024). However, broad‐spectrum antibiotics have resulted in the emergence of multidrug‐resistant (MDR) and extensively drug‐resistant (XDR) strains of *M. morganii*, leading to treatment failures in clinical settings (Karaiskos and Giamarellou 2014).

Silva LC et al. have advocated that this pathogen can no longer be ignored, given its increasing levels of virulence and antimicrobial resistance (Bandy 2020; Silva et al. 2024).

### 1.2 Re‐Shaping Taxonomy: New Insights for the *Morganella* Genus

This genus belongs to the order Enterobacterales and the family *Morganellaceae* (Farmer et al. 1985), and was previously divided into two species: *M. morganii* and *M. psychrotolerans*. Within *M. morganii*, two subspecies have been differentiated: *M. morganii* subsp. *morganii* and *M. morganii* subsp. *sibonii* (O'Hara et al. 2000).

A comprehensive genomic analysis was recently conducted on an international collection of *Morganella* spp. strains, leading to a proposed new taxonomy. *M. sibonii* has been considered as a separated species, and *M. morganii* has been split into two subspecies: *M. morganii* subsp. *morganii* and *M. morganii* subsp. *intermedius* (Bonnin et al. 2024).

The genome of *M. sibonii* showed significant differences from that of *M. morganii*, with specific characteristics such as a trehalose operon (present in *M. sibonii* and absent from *M. morganii*), distinct virulence factors (including specific type III and type VI secretion systems in the *M. sibonii* genome), and an intrinsic resistance gene (*tetD* involved in the resistance to tetracycline present only in the core genome of *M. sibonii*) (Bonnin et al. 2024). Unfortunately, no fast, accurate, and affordable method exists to differentiate *M. morganii* from *M. sibonii*. So far, the epidemiology and physiopathology of *Morganella* spp. remain poorly understood. Recent advancements in Matrix‐Assisted Laser Desorption/Ionization Time‐of‐Flight Mass Spectrometry (MALDI‐TOF MS) technology will be helpful to identify the clinical implications of both relevant clinical species (Duque et al. 2025).

### 1.3 Studying Secretion System Type VI Within *Morganell*a spp. Genome: A New Approach to Better Understand the Bacterium Physiopathology

There is a wide array of secretion systems (SS) ranging from type I to type XI. These systems may deliver proteins from the cytoplasm directly to target cells (T3SS, T4SS, and T6SS), to the extracellular space (T1SS, T2SS, two partner systems, and autotransporters), or to the periplasm (the Sec and Tat systems) (McQuade and Stock 2018).

Through extensive analysis of human gut genomes, Coyne et al. reported that T6SSs are widely present among Gram‐negatives, which represent approximately 25% of the bacteria in the human colon (Coyne et al. 2016). Individually, each bacterial isolate encodes between 1 and 6 distinct T6SS clusters (Boyer et al. 2009; Bingle et al. 2008).

In 2015, Li et al. proposed a classification scheme of T6SS based on the *tssB* gene sequence (Li et al. 2015). Four T6SS types (T6SSi‐iv) could then be identified. The canonical T6SSi, found mainly in Proteobacteria, is divided into six subtypes (i1, i2, i3, i4a, i4b, and i5). T6SSii and T6SSiii were found exclusively on *Francisella* spp. pathogenicity islands and *Bacteroidetes* spp., respectively; while T6SSiv was observed in *Amoebophilus* spp. (Li et al. 2015; Gallegos‐Monterrosa and Coulthurst 2021; Bayer‐Santos et al. 2019).

This contractile nanomolecular apparatus can deliver several toxins, also called effectors, that target the cell envelope of prokaryotic and/or eukaryotic cells. This system is involved in a wide range of functions, such as interbacterial killing and growth inhibition, nutrient scavenging, host colonization, kin discrimination, acquisition of genetic material, metal uptake, and antifungal activity (Gallegos‐Monterrosa and Coulthurst 2021; Bernal et al. 2017; Peñil‐Celis and Garcillán‐Barcia 2019; De Sousa et al. 2021; Han et al. 2019).

The genomic organization of T6SS is generally composed of 13 core conserved genes that encode structural proteins (TssA‐M): membrane complex (TssJML), baseplate (TssKEFG), needle spike (VgrG/TssI and PAAR), sheath (TssBC) (Cascales and Cambillau 2012), and a PAAR (for proline–alanine–alanine–arginine repeats) structural component that caps the VgrG/TssI spike. This nanomolecular weapon is activated in a contact‐dependent manner. The contraction of the extended sheath effector delivery involves sheath contraction that propels the Hcp‐VgrG‐PAAR structure into the target cell. After contraction, TssH ATPase‐mediated disassembly of the T6SS occurs and prepares the system for another triggering event (Basler et al. 2012; Wang et al. 2017; Filloux 2013; Shneider et al. 2013; Pukatzki et al. 2007; Bernal et al. 2021).

Two types of effectors can be delivered: cargo effectors, which interact non‐covalently with VgrG/Hcp/PAAR domains; and specialized effectors that possess a bifunctional domain, including one containing a VgrG/Hcp‐/PAAR domain (Coulthurst 2019; Durand et al. 2014). Accordingly, a wide diversity of effectors, such as nucleases, amidases, hydrolases, or phospholipases, have been reported to be delivered through T6SS (Durand et al. 2014; Russell et al. 2012; Tang et al. 2018; Wang et al. 2020; Whitney et al. 2013). The effectors encoding genes are mostly present within the T6SS clusters but can be localized in orphan islands elsewhere in the genome. They are commonly located downstream of *hcp*, *vgrG*, or *PAAR* genes. Genes encoding immunity proteins conferring resistance to effectors and protecting the secreting bacteria are most often localized just upstream or downstream the effector‐encoding genes (Navarro‐Monserrat and Taylor 2023a).

This study aimed to describe the distribution and diversity of type VI secretion systems (T6SS) among clinically relevant *Morganella* species: *M. morganii* and *M*. *sibonii*. Currently, knowledge regarding the prevalence and implications of each species in clinical settings remains limited. Although several virulence factors have been reported, no study has specifically examined the T6SS in *Morganella* species yet. Since T6SSs usually play a crucial role in the interactions of bacteria with their environment, the identification of a putative T6SS specific of *M. sibonii* has drawn attention to the study of T6SS in *Morganella* spp. (Bonnin et al. 2024). In this study, we characterized the T6SS clusters in a collection of 293 *Morganella* clinical isolates. Additionally, we conducted an extensive in silico characterization of the effectors within these T6SS clusters.

## 2 Material and Methods

### 2.1 Strains Collection

We retrospectively analyzed an international collection of 293 strains of *Morganella* isolates collected between 1996 and 2023. These strains were isolated in Germany (*n* = 32), the United Kingdom (*n* = 18), Austria (*n* = 2), Belgium (*n* = 24), Denmark (*n* = 2), Canada (*n* = 1), the Netherlands (*n* = 4), Poland (*n* = 1), and France (*n* = 58).

Since our first set (*n* = 141) of *Morganella* isolates mostly included multidrug‐resistant strains (Bonnin et al. 2024), 152 additional clinical isolates were collected from clinical isolates in Kremlin‐Bicêtre and Paul‐Brousse French tertiary hospitals from 2020 to 2023 to be more representative of the global epidemiology of *Morganella* spp. These isolates were cultured from urine (*n* = 49), digestive tract (*n* = 22), respiratory tract samples (*n* = 33), blood cultures (*n* = 21), and other various origins (*n* = 26).

### 2.2 Whole‐Genome Sequencing (WGS) and Phylogenetic Analysis

WGS was performed on all included isolates using Illumina technology. Reads were assembled using Shovill v1.1.0 and Spades v3.14.0. Resistome analysis was performed using the ResFinder server v3.0 (https://cge.cbs.dtu.dk/services/ResFinder/).

To assess population bias, we performed a core‐genome‐based phylogenetic analysis of our collection. Genome annotation was done with Prokka v1.14.6, and pan‐ and core‐genome identification with Panaroo v1.5.1 (70% protein identity for pangenome families, 80% presence for core genes). Core genome alignment used MAFFT v7.490. The best‐fit model, selected via Bayesian Information Criterion (BIC) in ModelFinder Plus, guided phylogenetic inference with IQ‐TREE v2.0.7. The final phylogenetic tree was visualized using iTOL v6.5.2.

To determine species and subspecies, we cross‐validated the Phylogeny with a hierarchically clustered ANI matrix in which we added reference genomes.

Six strains were sequenced using Nanopore long‐read technology, including two *M. sibonii* (O103E8 and CIP103649), three *M. morganii* subsp. *intermedius* (O104E2, O103I6, and O103J10), and one *M. morganii* subsp. *morganii* (O103F1).

### 2.3 Identification, Classification, and Visualization of T6SS Clusters

The SecReT6 web platform (https://bioinfo-mml.sjtu.edu.cn/SecReT6/phylogenetic_analysis.php) was used to annotate and predict the T6SS clusters. The T6SS prediction tool which detects T6SS gene clusters used BLASTp 2.10.1+ against experimentally validated T6SSs recorded in SecReT6. The dataset parameters used were: an e‐value threshold< 0.0001, a BLAST identity threshold for T6SS prediction > 30%, a BLAST Ha‐value threshold for T6SS‐related protein prediction > 0.42 (0–1), a maximum os 15,000 pb interval between two co‐localized T6SS components, and a minimum of three T6SS component proteins for each T6SS region. All the clusters annotated but not classified were curated manually by analyzing RAST annotated fasta file on CLC Genomics Workbench v10.0.1 (Qiagen, Les Ulis, France).

The classification of T6SSs was based on the TssB protein since it was observed that this protein alone may be a suitable classification marker (Boyer et al. 2009; Russell et al. 2013).

### 2.4 Comparison of T6SS Clusters Between Different Strains: Nucleotide Homology and Synteny

A representative strain of each *Morganella* species has been selected: *M. sibonii* O103E8, *M. morganii* subsp. *intermedius* O103I6, and *M. morganii* subsp. *morganii* O112F10.

All T6SS subtype clusters identified with SecReT6 were annotated with RAST, and compared using the online CompArative GEne Cluster Analysis Toolbox (CAGECAT) (https://cagecat.bioinformatics.nl). This tool provides homology searches and synteny comparison of the whole‐genome cluster using the cblaster and clinker pipelines.

#### 2.4.1 Phylogeny of *tssB* Genes

The *tssB* nucleotide sequence was extracted from SecReT6 annotation. All the *tssB* fasta files annotated were then analyzed with the multi‐sequence alignment tool: CLUSTALW. The phylogenetic tree of *tssB* genes was visualized using iTOL v6.5.2.

#### 2.4.2 In Silico Prediction of T6SS Effectors and Immunity Proteins

SecReT6 annotate effectors or immunity proteins but it relies on already known hits. To avoid any bias due to totally novel effector and immunity, we manually curated genetic environments of *vgrG* genes to detect effectors and their immunity proteins. Genomes sequenced by Illumina were annotated with Prokka (Seemann 2014). All *vgrG* genes annotated with Prokka were extracted, as well as upstream (approximately 15 genes) and downstream genes. The sequences were then translated using BlastP and tested using both the NCBI database (https://www.ncbi.nlm.nih.gov/index1.shtml) and Alphafold (https://alphafold.com/). The highest average score in the predicted local distance difference test (pLDDT) was recovered on Alphafold to detect non‐annotated effectors. The functional domain of the protein was determined by using the IPR or Pfam number.

### 2.5 Comparison of Nucleotide Sequences of the Most Prevalent T6SS Effectors

The nucleotide sequence of the genes encoding for TseV, Tle (lipase), Rhs, and S‐type pyocin was determined by using blastx, on four different *Morganella* spp. strains (*M. morganii* subsp. *morganii* 101J3: TseV; *M. sibonii* O112D5: *M. sibonii* S‐type pyocin; O103E8: Rhs; *M. morganii* subsp. *intermedius* 131E1: Tle). All similar encoding genes were found by using tblasn, and sequence alignment was done with MAFFT v7.490. The nucleotide distance of each determined by comparing all encoding similar genes present on a different contig and represent as a heatmap.

### 2.6 *Morganella* spp. Genomes

Genomes of *Morganella* spp.; isolates were previously deposited in the GenBank database in bioproject PRJNA996528 (Bingle et al. 2008).

## 3 Results

Our collection of 293 *Morganella* spp. clinical isolates included 228 *M. morganii* subsp. *morganii* (77.8%), 39 *M. morganii* subsp. *intermedius* (13.3%), and 26 *M. sibonii* (8.9%). All isolates were previously whole‐genome sequenced (WGS) (Bonnin et al. 2024).

This collection includes 104 carbapenemase producers. The produced carbapenemases were NDM‐1 (*n* = 47), NDM‐5 (*n* = 8), NDM‐7 (*n* = 3), OXA‐48 (*n* = 20), OXA‐204 (*n* = 1), OXA‐641 (*n* = 1), KPC‐2 (*n* = 19), VIM‐1 (*n* = 2), IMP‐27 (*n* = 1), NDM‐1 + KPC‐2 (*n* = 1), and NDM‐1 + OXA‐48 (*n* = 1) (Supporting Table 1).

Using SecReT6 v3 we investigated the prevalence and distribution of T6SS clusters in the 293 genomes of *Morganella* spp. Approximately half of the strains (150/293, 51.2%) harbored at least one cluster within their genome. Of note, the ratio of T6SS‐harboring *Morganella* spp. was higher (but not significant, *p* = 0.16) among carbapenemase producers 59/104 (56.7%) compared to non‐carbapenemase producers 91/189 (48.1%) (Supporting Table 1).

### 3.1 Diversity of T6SS Subtypes Among *Morganella* Species

**FIGURE 1:**
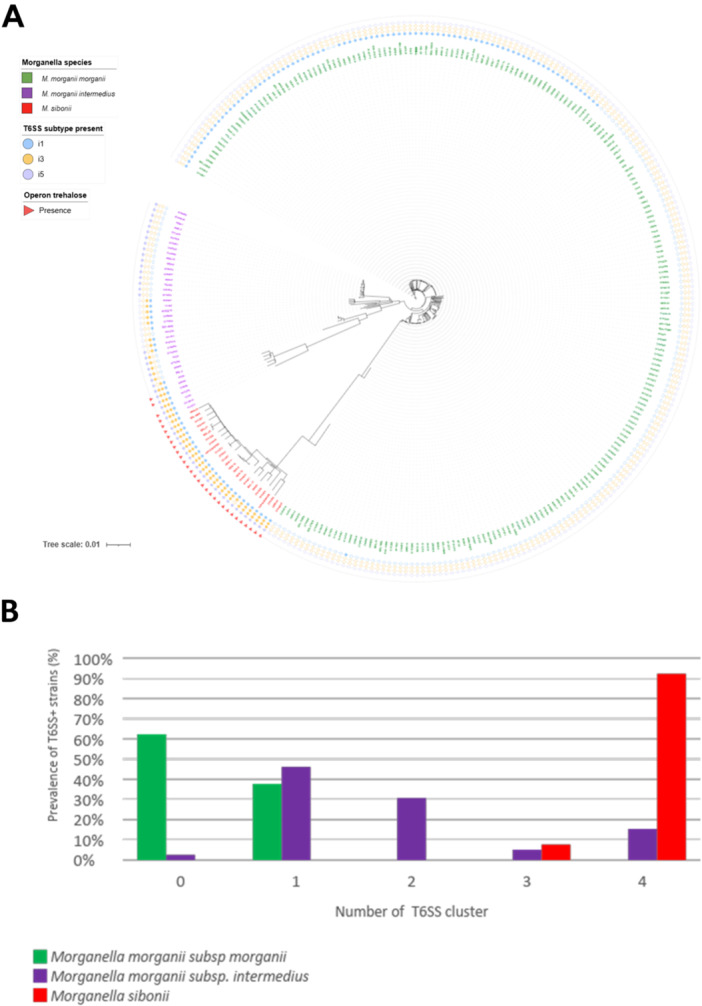
(A) Phylogenetic tree of *Morganella* spp. and distribution of T6SS cluster, subtypes, and presence of trehalose operon. (B) Prevalence (%) of T6SS+ isolates according to T6SS number of clusters among *M. morganii* subsp. *morganii*, *M. morganii* subsp. *intermedius*, and *M. sibonii*.

**FIGURE 2:**
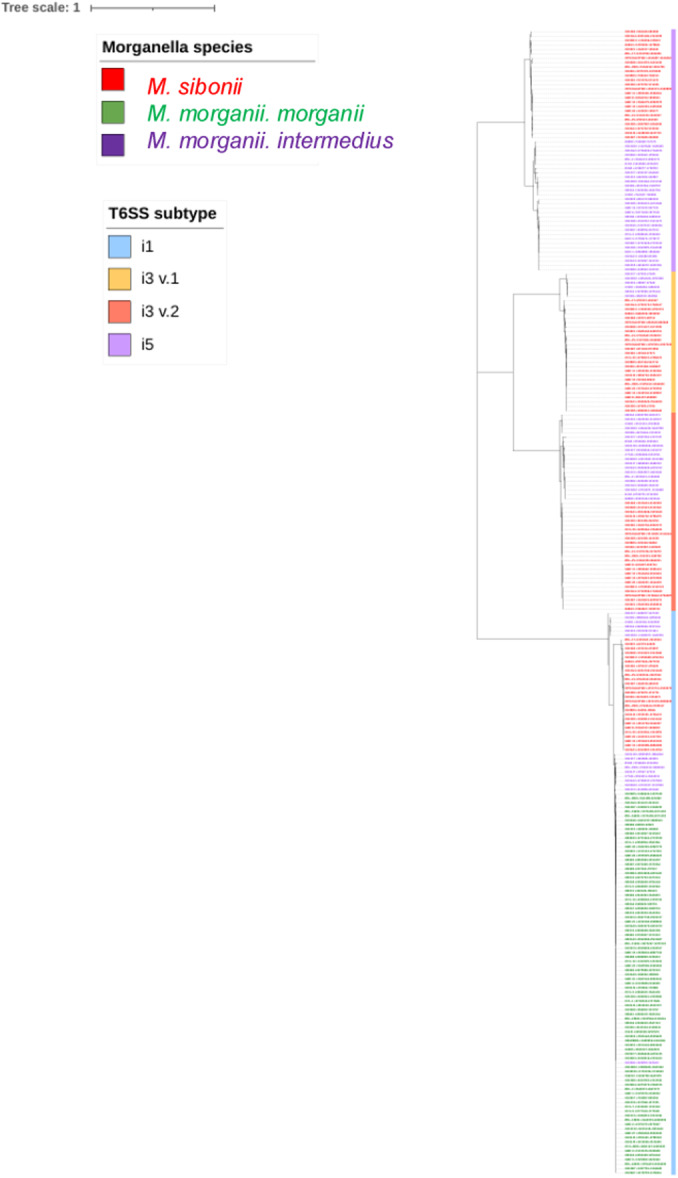
Phylogenetic tree of *tssB* genes annotated with SecreT6 and generated with CLUSTAL and iTOL, related to its T6SS subtype. Orange: T6SS i3 versions identified among *M. sibonii* and *M. morganii* subsp. *intermedius*: i3 v.1 (operon trehalose positive isolates), and i3 v.2. Blue: T6SS i1 subtype, identified among all *Morganella* spp. Purple: i5 subtype, predominantly within *M. morganii* subsp. *intermedius* and *M. sibonii*.

The different T6SS of *Morganella* spp. were classified according to the TssB amino acid sequence and categorized as “i” subtypes, as previously reported in the SecReT6 database for other Proteobacteria and other bacterial species (Li et al. 2015). Accordingly, three major subtypes have been identified: i1, i3, and i5 (Figures 1A and 2). The most prevalent combination of T6SS subtypes was: i1, i3v.1, i3v.2, and i5 in *M. sibonii* (92%); i1 in *M. morganii* subsp. *morganii* (37.7%), and i5 in *M. morganii* subsp. *intermedius* (46%) (Figure 1B).

It is noteworthy that *M. sibonii* exhibited at least three T6SS clusters (7.7%%, 2/26) or four T6SS clusters (92.3%, 24/26). Conversely, 62.3% (142/228) of *M. morganii* subsp. *morganii* isolates did not contain any T6SS clusters in their genome, and 37.7% (86/228) contained only one T6SS cluster, systematically belonging to the subtype

i1. Interestingly, the recently identified *M. morganii* subsp. *intermedius* contained a variable number of clusters, from zero (1/38 isolates) to four (15%, 6/39). Half of these *M. morganii intermedius* isolates (46%, 18/39) contained only one T6SS belonging to i5 subtype (Figure 1). Both *M. morganii* subsp*. morganii* and *M. morganii* subsp. *intermedius* could be differentiated on their single T6SS cluster: type i1 and i5, respectively.

**FIGURE 3:**
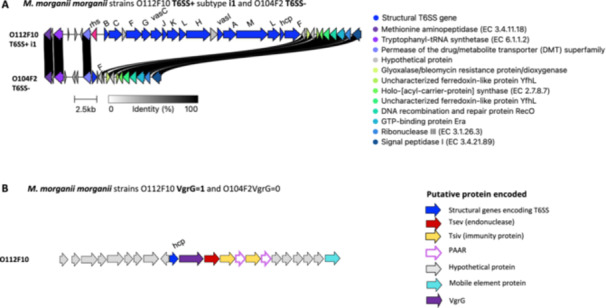
(A) Schematic representation of intact T6SS locus i1 subtype of *M. morganii* subsp. *morganii* strain O112F10, compared to the T6SS negative *M. morganii* subsp. *morganii* strain O104F2, retaining only the *tssF* gene in the gene loci. Dark blue arrows represent structural gene of T6SS cluster *tss*; gray arrows, hypothetical protein; red arrow, *tseV* (putative effector encoding gene), yellow arrow, *tsiV* (putative immunity protein‐encoding gen); and pink arrow PAAR domain proteins encoding genes. The schematic representation of the genetic environments of T6SS clusters was obtained using RAST annotation, and the comparison of the two T6SS clusters of O112F10 and O104F2 was performed by using CAGECAT bioinformatic tool. The annotation of the putative functions encoded by the genes downstream of the VgrGs was obtained using the BlastP tool and compared with the NCBI and Alphafold databases. (B) Representation of the genetic environment of an orphan *vgrG* (strain O112F10), located upstream *tseV* and *tsiV* genes encoding TseV endonuclease (its putative effector), and its related cognate immunity protein TsiV, predominantly present among *M. morganii* subsp. *morganii*.

The synteny of T6SS clusters was conserved among *Morganella* species (Supporting Figure 1). Indeed, although subtype i1 was the unique present in *M. morganii* subsp. *morganii* T6SS+, it was also present in the other *Morganella* species (Figure 3A, Supporting Figure 1). Of note, the analysis close genetic context of the T6SS i1 cluster of the *M. morganii* subsp. *morganii* identified the presence of *tssF* gene in T6SS‐negative isolates of *M. morganii* subsp. *morganii* (Figure 3A), suggesting a partial deletion of this T6SS i1 cluster in T6SS negative isolates.

**FIGURE 4:**
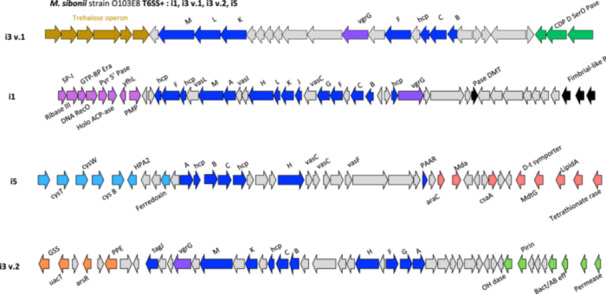
Schematic representation of intact T6SS i1, i3 v.1, i3 v.2, and i5 subtypes of *M. sibonii* strain O103E8. Each cluster has a different composition of structural genes (*tss*), some of them are specifically found in one subtype, for example, *tssJ* in subtype i1. The schematic representation of the genetic environments of T6SS clusters was obtained using RAST annotation. The annotation of the putative functions encoded by the genes downstream of the VgrGs was obtained using the BlastP tool and compared with the NCBI and Alphafold databases. * CDP D SerO Pase: CDP diacylglycerol serine O phosphatidyltransferase; SP‐I: Signal peptidase I; Ribase III: Ribonuclease II; GTP‐BP Era: GTP‐binding protein Era; DNA RecO: DNA recombination and repair protein RecO; Pyr 5′ Pase: Pyridoxine 5′ phosphate synthase; Holo ACP‐ase: Holo acyl‐carrier‐protein synthase; yfhL: Uncharacterized ferredoxin like protein YfhL; PMP: probable membrane protein; Pase DMT: Permease of the drug/metabolite transporter (DMT) superfamily; Fimbrial‐like P: putative fimbrial‐like protein; cyst: Sulfate transport system permease protein CysT; cysW: Sulfate transport system permease protein CysW; Sulfate and thiosulfate import ATP‐binding; cys B: Cysteine synthase B; HPA2: Histone acetyltransferase HPA2; araC: Transcriptionnal regulator AraC; Mda: modulator of the drug activity; csaA: Protein secretion chaperonin CsaA; D‐t symporter: d‐tripeptide/cation symporter; MdtG: Multidrug resistance protein MdtG; LipidA: LipidA biosynthesis lauroyl acyltransferase; Tetrathionate rase: Tetrathionate reductase two‐component; GSS: Glutathionyl spermidine synthase; uacT: Uric acid proton transporter UacT; arsR: Transcriptional regulator ArsR family; PPE: PPE family protein; OH dase: Alcool deshydrogenase; Bact/AB eff: Bacteriocin/antibiotic efflux ABC transporter; Permease: Putative secretion permease.

Regarding *M. sibonii* most of the isolates harbored four clusters belonging to subtypes i1, two distinct versions of the subtype i3 (i3 v.1 and i3 v.2) and the subtype i5 (Figures 1 and 2). The i3 v.1 cluster, identified just downstream of the trehalose operon, was specific of *M. sibonii* (Figure 4). Conversely, the T6SS i3 v.2 cluster was also identified in *M. morganii* subsp. *intermedius* (Figure 2, Supporting Figure 1). A comparison of the two versions of i3 present simultaneously in *M. sibonii* (e.g., strain O103E8) showed that some *tss* genes, such as *tssA, tssG*, or *tssH* are present in i3 v.2 but absent in i3 v.1 (Figure 4). In addition, as shown in Figure 2, *tssB* of T6SS i3 v.1 and *tssB* of T6SS i3 v.2 are phylogenetically distant. These results are in favor of the acquisition of two independent clusters at distinct locations in the genome rather than a duplication of one T6SS i3 in *M. sibonii*.

### 3.2 Prevalence and Diversity of the *vgrG* Genes in *Morganella* Species

Since it is well known that VgrG effectors might be encoded by orphan genes outside the T6SS clusters, (Navarro‐Monserrat and Taylor 2023b) *vgrG* genes were searched on the whole genomes and annotated using Prokka (Supporting Table 2).

Regarding the 228 isolates of *Morganella morganii* subsp. *morganii*, only 123 *vgrG* (54.0%, 123/228) were identified, including 80 T6SS i1 positive isolates (35.1%, 80/228) and 31 T6SS negative strains (13.6%, 31/228). Of note, six T6SS i1 positive isolates (2.6%, 6/228) possessed two *vgrG* genes localized on different contigs. For these six last strains, the short read sequencing, could not differentiate a true duplication of the gene or a truncated *vgrG* gene that would appear on two different contigs.

In contrast, 71 *vgrG* were annotated among *M. sibonii* genomes, all strains were T6SS+, and possessed between one and six *vgrG*. Three isolates possessed one *vgrG* (11.5%, 3/26) seven isolates harbored two *vgrG* (26.9%, 7/26), 12 had three *vgrG* (46.2%, 12/26), three had four *vgrG* (11.5%, 3/26), and one possessed six *vgrG* (3.9%, 1/26).

Over the 39 isolates of *M. morganii* subsp *intermedius*, 38 isolates were T6SS+ and harbored zero to seven *vgrG* (zero *vgrG*: 56.4%, 22/39; one *vgrG*: 5.1%, 2/39; two *vgrG*: 5.1%, 2/39; three *vgrG*: 2.6%, 1/39; four *vgrG*: 18.0%, 7/39; five *vgrG*: 5.1%, 2/39; six *vgrG*: 2.6%, 1/39; seven *vgrG*: 2.6%, 1/39). Only one strain was T6SS negative and possessed one *vgrG* gene. Interestingly, all *M. morganii* subsp *intermedius* containing one T6SS i5 subtype have no *vgrG* annotated with Prokka in their genome. (Supporting Table 2).

### 3.3 Effectors and Immunity Proteins in *Morganella* spp.

Among *M. morganii* subsp. *morganii* genomes TseV encoding gene (encoding a putative endonuclease) was widely represented (95%, 117/123 effectors) (Table 1). This *tseV* gene was followed by *tsiV*, encoding its putative immunity protein TsiV (Figure 3B). Two other putative effectors have been identified in *M. morganii* subsp. *morganii*: a putative lipase (4%; 5/123) and a RhS‐type protein (0.8%,1/123). Of note, since these two effectors likely target eukaryotic cells, we did not identify any immunity protein‐encoding genes (Table 1, Supporting Figure 2).

**TABLE 1:** Prevalence of the effectors and immunity proteins among *Morganella* species. Domain and putative function were described for each putative T6SS putative effector, immunity proteins, and miscellaneous proteins. For each putative effector, an example of the same or similar effector already characterized T6SS effector in the literature was given, as well as the associated organisms, targeted cells, and references. * Indicates that the putative encoding protein could be duplicated within the same genome.

Putative effector	Domain	Putative function	M. morganii*	M. intermedius*	M. sibonii*	Total number*	Example of same or similar effector delivered by T6SS and already caracterized	Target cell	Biochemical activity	Associated organism	Reference
TseV*	VRR NUC domain: PF08774	Endonuclease	117	8	10	135	TseV	Antibacterial	Endonuclease	*P. aeruginosa; S. bongori*	(Hespanhol et al. (2022); Wang et al. (2021))
Lipase*	Lipase family protein	Lipase	5	10	26	41	Tle 1‐5; TseL	Transkingdom effector (eucaryote and procaryote)	Cause membrane damage	*P. aeruginosa; V. cholerae*	(Jiang et al. (2016); 2014; Dong et al. (2013))
RHS repeat‐associated core domain‐containing protein/DUF6531/endoU*	PF20148	Endonuclease	1	28	10	39	RhsA/RhsB	Antibacterial (ex: *E. coli*)	NS‐2 endonuclease/HNH endonuclease	*Dickeya dadantii*	(Koskiniemi et al. (2013))
Polymorphic toxin type 33 domain containing protein	Toxin type 33: PF1553	RNase	0	0	1	1	Ntox33 delivered by T2SS	Antibacterial	Predicted RNase toxins with uncertain metal dependence	Actinobacteria, cyanobacteria, firmicutes, γ‐proteobacteria, verrucomicrobia	(Zhang et al. (2012b))
Polymorphic toxin type 15/Tde1	PF15604 Novel toxin 15/Tde1	RNAse/DNAse	0	1	1	2	Tde1/Ntox15	Antibacterial	DNA fragmentation through	*Bacteroidales*	(Bosch et al. (2023))
S type pyocin*	PF06958	Endonuclease	0	0	13	13	S‐type pyocin	Antibacterial	DNase dependent on Mg2+, Ni2+, Mn2+, and Co2+ bivalent metal ions	*Y. pseudotuberculosis*	(Yang et al. (2024b))
Toxin VasX*	PF20249	Compromise the inner membrane of prokaryotic target cells	0	7	0	7	VasX	Anti‐eucaryotic effector *Dictyostelium discoideum* (ameba)	Membrane depolarization leading to pore formation	*V. cholerae*	(Miyata et al. (2011b))
DUF4150	Toxin PAAR‐like domain (Tse7), PF13665	DNAse	0	1	6	7	Tse7	Antibacterial	DNAse	*P. aeruginosa*	(Pissaridou et al. (2018))
Type II toxin/antitoxin system RelE/ParE	IPR035093	Ribosome‐dependent mRNA endoribonuclease which inhibits translation during amino acid starvation	0	0	1	1	ParE	Antibacterial (*E. coli*)	Inhibition early stages of DNA replication	Wide range of Gram−	(Jiang et al. (2002a))

T6SS accessory proteins or adaptator	Domains	Putative functions	M. morganii*	M. intermedius*	M. sibonii*	Total number*	Family protein	Example of loading protein	Associated organism	Reference
DUF4123*	DUF4123	Unknown	1	31	53	85	Tec/Tap	TseL	*V. cholerae*	(Liang et al. (2015))
DUF2169*	DUF2169	Unknown	1	5	7	13	Atu3641	Tde2	*A. tumefaceins*	(Bondage et al. (2016))
DcrB‐related protein	DUF1795/PF08786	Phages C1 and C6 adsorption	0	11	1	12	Eag	Specialized PAAR effectors	*S. marcesens, P. aeruginosa*	(Cianfanelli et al. (2016); Whitney et al. (2015); Alcoforado Diniz and Coulthurst (2015))

Putative immunity protein	Domain	Putative functions	M. morganii*	M. intermedius*	M. sibonii*	Total number*	Immunity paired form with toxin	Reference
TsiV*	Type VI immunity for VRR NUC domain: PF11876	Type VI immunity family protein, cognate antitoxin of TseV	154	9	9	172	TseV	(Wang et al. (2021))
ParD‐like family protein	PF09386	Cognate antitoxin of ParE	0	0	1	1	ParE	(Jiang et al. (2002b))
Tdi1*	GAD‐like/DUF1851: PF02938/PF08906	Unknown/Type VI Immunity protein, cognate antitoxin of Tde1	0	5	2	7	Tde1/Ntox15	(Bosch et al. (2023); Ma et al. (2014b))
SMI1/KNR4 family protein (SUKH superfamily)*	IPR037883	Immunity protein	0	3	5	8	DNases (HNH/EndoVII‐ and restriction endonuclease‐fold, and RNases of the EndoU‐like and colicin E3‐like cyto toxic RNases‐folds)	(Zhang et al. (2011))

Miscellaneous	Domains	Putative functions	M. morganii*	M. intermedius*	M. sibonii*	Total number*
Integrase domain‐containing protein	PF12835	Integrase DNA‐binding protein	9	1	5	15
STM0539 family protein	NF033820	Unknown	0	10	0	10
CPBP family intramembrane glutamic endopeptidase	PF02517	Metalloendopeptidase activity/CAAX‐box protein processing	0	2	0	2
CPBP family intramembrane metalloprotease	PF02517	Metalloendopeptidase activity/CAAX‐box protein processing	0	2	0	2
DMSO/selenate family reductase complex A subunit	TIGR02166	Dimethyl sulfoxide reductase	1	0	0	1
ParA family protein	IPR050678	Partitioning	0	1	0	1
Methyl‐accepting chemotaxis protein III	PF00015	Receptor/transducer	0	3	0	3
Antirestriction protein ArdA	PF07275	ArdA functions in bacterial conjugation to allow an unmodified plasmid to evade restriction in the recipient bacterium and yet acquire cognate modification	0	2	0	2
Glycosyltransferase	Glycosyltransferase	Glycosyltransferase	0	1	0	1

Other	Domains	Putative functions	M. morganii*	M. intermedius*	M. sibonii*	Total number*
PAAR*	PF05488	Unknown	152	21	42	215
DUF932	DUF932	Unknown	0	1	0	1
Helix‐turn‐helix domain‐containing protein	PF01381	DNA‐binding regulator	0	1	0	1

Within *M. sibonii*, the most commonly identified effectors likely target eukaryotic cell with a putative lipase (35%, 26/74 effectors) and S‐type pyocin (13.5%, 13/74 effectors) (Table 1). Endonucleases such as TseV or RhS‐type proteins were less prevalent (13.5%; 10/74) in *M. sibonii* compared to *M. morganii* subsp. *morganii*. Few effectors, such as polymorphic toxin type 33 (RNase) or toxin/antitoxin systems (Tde1/Tdi1 or ParE/ParD) have been identified in only one isolate of *M. sibonii* (Table 1, Supporting Figure 2).

According to their phylogenetic position between *M. morganii* subsp. *morganii* and *M. sibonii*, *M. morganii* subsp. *intermedius* isolates displayed a combination of effectors identified in both species, including Rhs repeat‐associated core domain‐containing protein (28/57; 49.1%), lipase (10/57; 17.5%), TseV (8/57, 14.0%), and Tde1 (1/57, 17.5%). The VasX toxin (absent from *M. morganii* subsp. *morganii* and *M. sibonii*) was identified in 12.3% (7/57) of the *M. morganii* subsp. *intermedius* isolates. The Tde1, glycosyltransferase, and DUF4150‐containing proteins are frequently encountered (Table 1).

Moreover, the nucleotide distance of the most prevalent effectors was determined among *Morganella* spp. and presented in Supporting Figures 3–6. Of note, the total number of the encoding effector gene found by searching tblastn was higher than the number obtained by the method searching for effectors downstream of *vgrG* since the whole genome was analyzed using tblastn searching.

Comparison of the *tseV* nucleotide sequences identified low nucleotide distances ranging from 0% to 35%. Two distinct highly conserved *tseV*‐like genes, named: *tseV*_M1 and *tseV*_M2, were identified. The *tseV*_M1‐like gene was mostly presented in *M. morganii* isolates, and displayed 95%–100% identity. The *tseV*_M2‐like gene was predominantly found among *M. sibonii* genomes, harboring 88%–100% of homology (Supporting Figure 3).

The *rhs*, *tle* (lipase), and *s‐type pyocin* encoding effectors displayed lower nucleotide identities of 67%, 51%, and 52%, respectively (Supporting Figures 4–6).

The *rhs*‐like gene mostly annotated among *M. sibonii* strains seemed to cluster in two major distinct groups: the *rhs*_M1 gene (71% homology) and the *rhs*_M2 gene (88% homology) (Supporting Figure 4).

The *tle*_M1‐like encoding a lipase have been found in all *Morganella* spp. and shared a minimum of 85% identity, whereas *tle*_M2‐like, *tle*_M3‐like, and *tle*_M4‐like only presented within *M. morganii* subsp*. intermedius* and *M. sibonii* strains, displayed 97%, 88%, and 92% homology sequence, respectively (Supporting Figure 5).

For the *s‐type pyocin* encoding effector, all *M. sibonii* strains carried a *s‐type pyocin‐*like gene, called: *s‐type pyocin*_M1 (with a minimum of 68% of homology) (Supporting Figure 6).

## 4 Discussion

This study represents the first large‐scale genomic analysis investigating the distribution and prevalence of T6SS within the *Morganella* genus. We found that 51.2% (150/293) of the isolates harbored at least one T6SS cluster, highlighting the widespread presence of this system in *Morganella* spp.

Multidrug‐resistant isolates tended to carry more T6SS clusters than antibiotic‐susceptible isolates (56.7% vs. 48.1%), although this difference was not statistically significant (*p* = 0.16). The association between bacterial competition—partly mediated by T6SS—and antimicrobial resistance has been previously described (Hespanhol et al. 2023a). Indeed, DNA‐damaging effectors delivered during interbacterial competition may promote mutagenesis and contribute to the emergence of resistance. For instance, the double‐stranded DNA deaminase DddA from *Burkholderia cenocepacia* increases mutation rates leading to rifampicin resistance via mutations in the *rpoB* gene (De Moraes et al. 2021). Additionally, antibacterial effectors such as lipases or peptidoglycan hydrolases can induce target cell lysis, releasing extracellular DNA that may subsequently be acquired and integrated by competing bacteria, including resistance determinants (Cooper et al. 2017; Ringel et al. 2017; Borgeaud et al. 2015).

We identified up to four T6SS clusters per genome, distributed among three subtypes (i1, i3, and i5). Some subtypes appeared species‐specific, such as i1 in *M. morganii* subsp. *morganii* and i5 in *M. morganii* subsp. *intermedius*. Despite this diversity, the synteny of structural genes was conserved, and clusters were consistently located at the same chromosomal loci (Supporting Figure 1). However, recombination events leading to gain or loss of clusters were observed. For example, in most T6SS‐negative *M. morganii* subsp. *morganii* isolates, only the *tssF* gene remained, suggesting partial deletion of the ancestral i1 cluster (e.g., strain O104F2, Figure 3A).

A notable accumulation of diverse T6SS subtypes (i1, i3.v1, i3.v2, and i5) was observed in *M. sibonii*, raising questions about their functional specialization and target specificity. Given the shared evolutionary origin of *Morganella* species, niche‐specific selective pressures may have driven the retention and diversification of these systems. Furthermore, as reported in other bacteria (Hespanhol et al. 2023b), distinct T6SS subtypes are likely differentially expressed depending on environmental conditions.

In *M. morganii* subsp. *intermedius*, the presence of VgrG proteins (ranging from one to seven copies) and associated effectors correlated with isolates carrying both T6SS i1 and i3.v2. In contrast, no *vgrG* genes were identified in isolates harboring only T6SS i5 or the combination of i5 and i3.v2, raising questions about their functional capacity for effector delivery. Most TseV, lipase, and Rhs effectors were associated with T6SS i1‐positive isolates, suggesting that this subtype is primarily responsible for their secretion. Similarly, effectors shared between *M. sibonii* and T6SS i1/i5‐positive *M. morganii* subsp. *intermedius* (e.g., Tde1, DUF4150, and VasX) were consistently linked to T6SS i1, supporting a central role of this subtype in effector delivery.

The diversity of T6SS clusters in *M. sibonii* and *M. morganii* subsp. *intermedius* was mirrored by a broader effector repertoire compared to *M. morganii* subsp. *morganii*. Such diversification may confer a competitive advantage by limiting the emergence of resistance among competing bacteria (Smith et al. 2025).

Three effector families—TseV, lipases (Tle), and Rhs—were conserved across all *Morganella* species. TseV belongs to the PD‐(D/E)xK phosphodiesterase superfamily and contains a VRR‐Nuc domain involved in DNA metabolism. Similar effectors have been described in *Pseudomonas aeruginosa* (Wang et al. 2021) and shown to induce DNA double‐strand breaks and SOS response activation (Hespanhol et al. 2022). TseV forms an effector–immunity pair with TsiV, which was frequently duplicated and separated by a PAAR‐encoding gene. Interestingly, the conservation of the *vgrG–tseV–tsiV* locus in T6SS‐negative isolates suggests a retained immunity function rather than offensive capability.

Lipase effectors (Tle), identified in all species, act as trans‐kingdom toxins capable of degrading bacterial membranes and targeting eukaryotic cells to promote invasion or autophagy (Jiang et al. 2016, 2014). The absence of detectable immunity proteins supports a potential role in eukaryotic interactions (Zhang et al. 2012a). Similarly, S‐type pyocins identified in *M. sibonii* lacked associated immunity proteins and may function in both intra‐ and inter‐species interactions, as described in *Yersinia pseudotuberculosis* (Yang et al. 2024a). This effector was present in nearly half of *M. sibonii* isolates, suggesting species specificity, although further characterization is required.

Additional strain‐specific effectors were identified, including a type 33 toxin in *M. sibonii* strain 268G3 and a RelE/ParE toxin–antitoxin system in *M. sibonii* CIP103649. DNase effectors such as Tde (with Tdi immunity) shared homology with *Agrobacterium tumefaciens* and are known to mediate both interbacterial competition and niche colonization (Ma et al. 2014a). In *M. morganii* subsp. *intermedius*, effectors such as VasX and DUF4150 were associated with membrane permeabilization and virulence‐related functions (Miyata et al. 2011a).

Chaperone (adapter) proteins required for effector secretion were also identified. These included members of the Eag family (containing a DcrB/DUF1795 domain), which mediate interactions between VgrG and PAAR‐containing effectors, and DUF2169‐containing chaperones associated with Rhs and DUF4150 secretion. Notably, these chaperones were predominantly found in *M. morganii* subsp. *intermedius* and *M. sibonii*.

### 4.1 Study Limitations

This study has several limitations. First, although SecReT6 v3 is currently the most comprehensive tool for T6SS annotation, its database remains incomplete and relies on homology with previously characterized systems, potentially limiting the identification of novel effectors. To mitigate this, we complemented our analysis using BLASTp against broader databases such as NCBI and AlphaFold.

Second, most genomes were sequenced using short‐read technologies, which may introduce assembly artifacts. For instance, genes such as *tssB* may be truncated or misannotated, and adjacent contigs may be incorrectly merged into a single cluster. To address this, annotations were systematically cross‐checked with RAST. Nevertheless, long‐read sequencing technologies (e.g., Oxford Nanopore) would improve cluster resolution.

Finally, discrepancies were observed in the annotation of *vgrG* genes between Prokka and RAST, with Prokka often identifying orphan genes. Although RAST appeared more suitable for this analysis, methodological differences may have led to an incomplete characterization of the *vgrG* repertoire. Additionally, sequence analyses revealed high polymorphism among conserved genes, highlighting the complexity of effector annotation. Despite these limitations, this study provides a comprehensive overview of T6SS diversity and identifies numerous candidate effectors in *Morganella* spp.

## 5 Conclusion

Our analysis highlights novel effector candidates that might participate in bacterial warfare, adaptation, and dominance of *Morganella* species in some niches (anti‐bacterial) but also that might participate in pathogenesis with hosts (anti‐eukaryotic). These findings may pave the way for future research and offer new insights into our understanding of the *Morganella* genus pathogenicity. However, these results must be confirmed through wet‐lab experiments. It is crucial to characterize the implications of the T6SS in both species, clarify their role and mode of activation, and identify the functions and targets of the potential effectors, along with their corresponding immunity proteins.

## Author Contributions

**Mathilde Duque:** investigation, writing – original draft, visualization, formal analysis. **Aymeric Jacquemin:** software, formal analysis, investigation. **Delphine Girlich:** investigation. **Rémy A. Bonnin:** writing – review and editing, methodology. **Laurent Dortet:** writing – review and editing.

## Ethics Statement

The authors have nothing to report.

## Conflicts of Interest

The authors declare no conflicts of interest.

## Supporting information

Supporting File 1

Supporting File 2

Supporting File 3

Supporting File 4

Supporting File 5

Supporting File 6

Supporting File 6

Supporting File 8

## Data Availability

The data that support the findings of this study are available from the corresponding author upon reasonable request.
