# Supplementary material for: Genome‐Wide In Silico Analysis of the Type VI Secretion System (T6SS) Within the Morganella Genus
Source: Microbiologyopen. 2026 Apr 30;15(3):e70304. doi: 10.1002/mbo3.70304 (PMC13129497; doi:10.1002/mbo3.70304)
Supplement: Supplementary file 3 — Supporting File 3 [file MBO3-15-e70304-s001.pptx]

## Slide 1
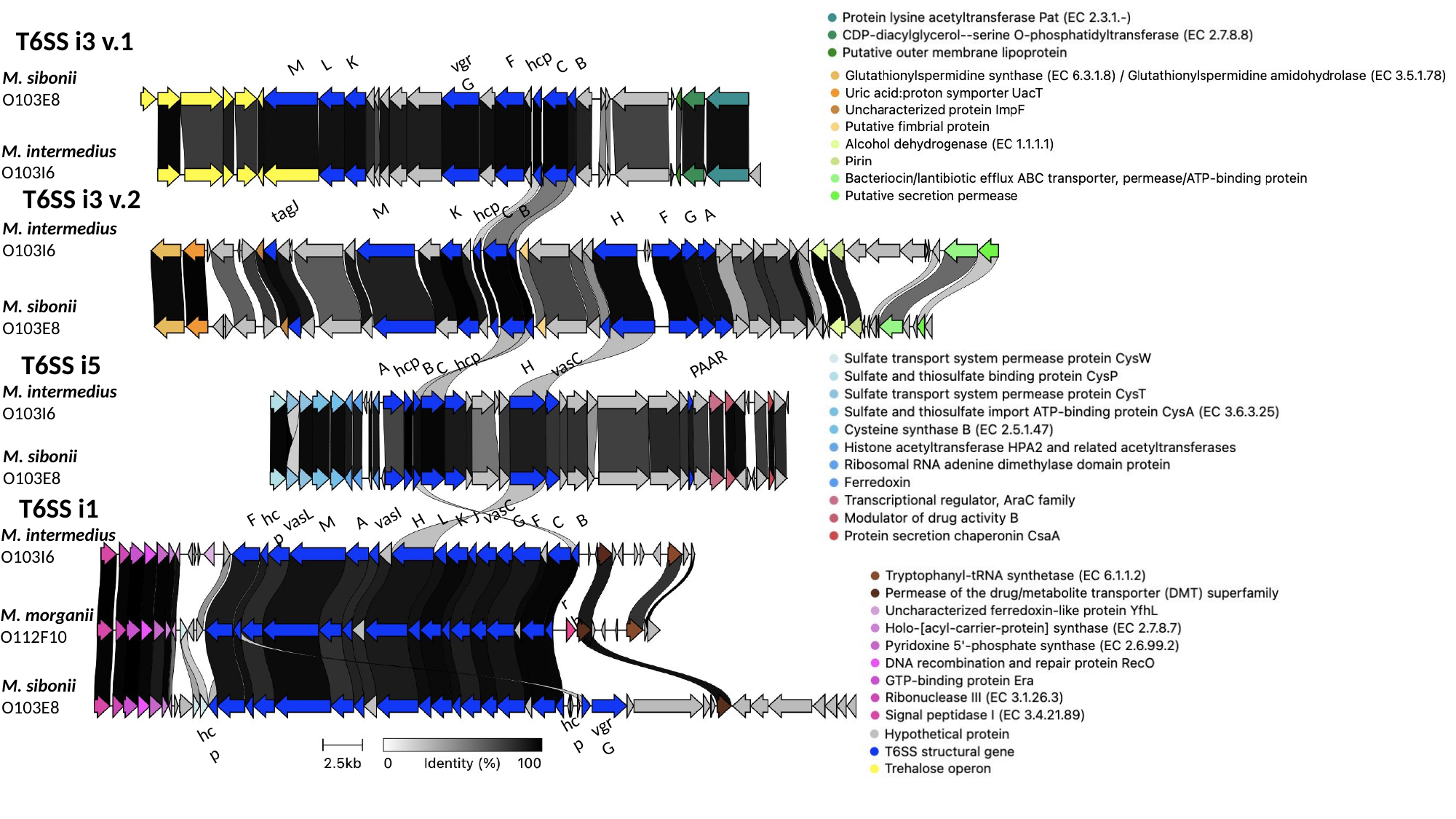

T6SS i3 v.1
hcp
vgrG
K
C
L
F
M
B
tagJ
hcp
M
B
K
C
A
F
G
H
PAAR
hcp
vasC
hcp
B
C
A
H
J
vasC
L
K
H
A
vasI
vasL
M
hcp
B
F
G
C
F
rhs
vgrG
hcp
hcp
M. sibonii
O103E8
M. intermedius
O103I6
T6SS i3 v.2
M. intermedius
O103I6
M. sibonii
O103E8
T6SS i5
M. intermedius
O103I6
M. sibonii
O103E8
T6SS i1
M. intermedius
O103I6
M. morganii
O112F10
M. sibonii
O103E8
