# Supplementary material for: Genome‐Wide In Silico Analysis of the Type VI Secretion System (T6SS) Within the Morganella Genus
Source: Microbiologyopen. 2026 Apr 30;15(3):e70304. doi: 10.1002/mbo3.70304 (PMC13129497; doi:10.1002/mbo3.70304)
Supplement: Supplementary file 4 — Supporting File 4 [file MBO3-15-e70304-s004.pptx]

## Slide 1
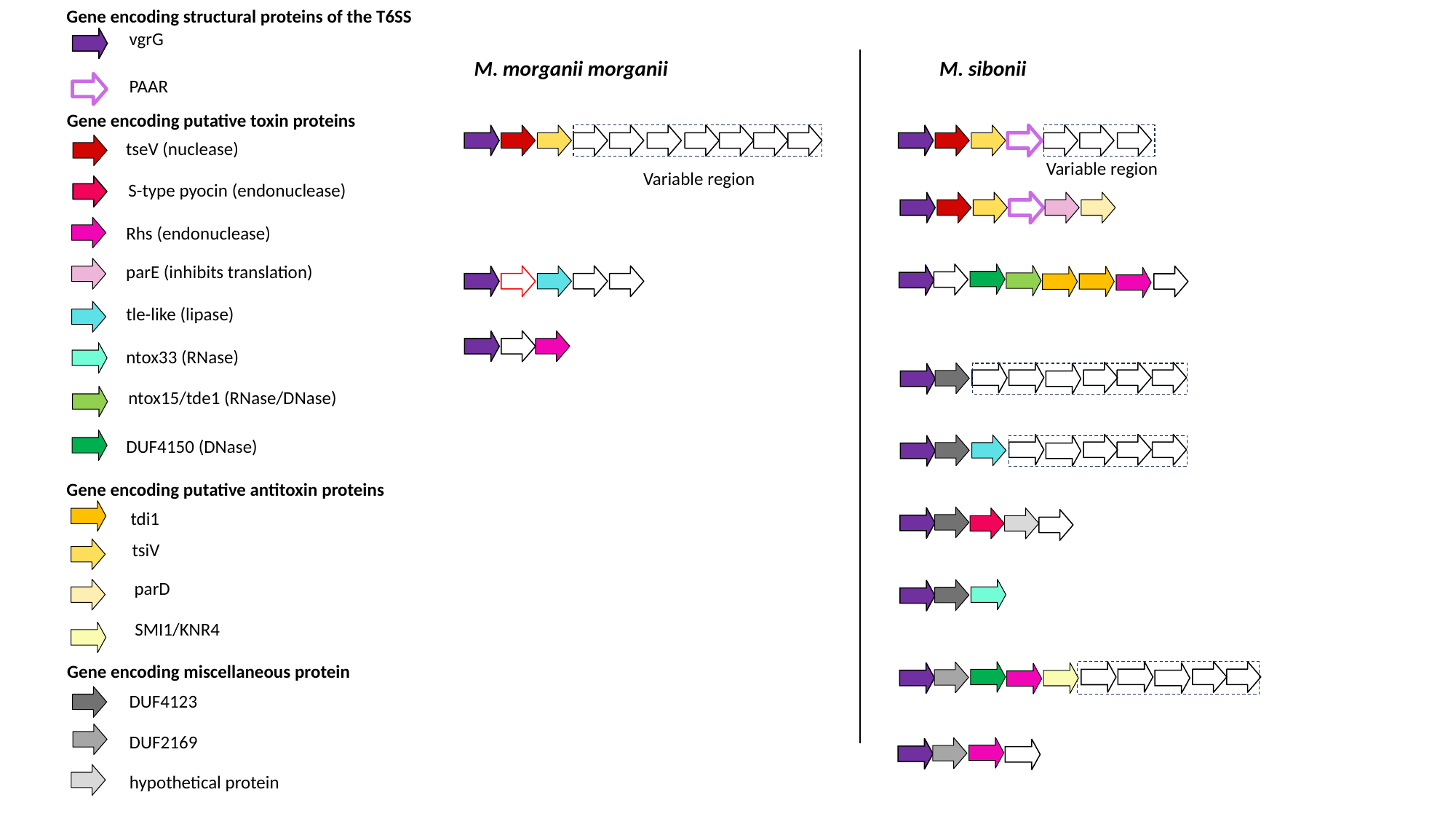

Gene encoding structural proteins of the T6SS
vgrG
M. morganii morganii
M. sibonii
PAAR
Gene encoding putative toxin proteins
tseV (nuclease)
Variable region
Variable region
S-type pyocin (endonuclease)
Rhs (endonuclease)
parE (inhibits translation)
tle-like (lipase)
ntox33 (RNase)
ntox15/tde1 (RNase/DNase)
DUF4150 (DNase)
Gene encoding putative antitoxin proteins
tdi1
tsiV
parD
SMI1/KNR4
Gene encoding miscellaneous protein
DUF4123
DUF2169
hypothetical protein
