# Supplementary material for: Genome‐Wide In Silico Analysis of the Type VI Secretion System (T6SS) Within the Morganella Genus
Source: Microbiologyopen. 2026 Apr 30;15(3):e70304. doi: 10.1002/mbo3.70304 (PMC13129497; doi:10.1002/mbo3.70304)
Supplement: Supplementary file 5 — Supporting File 5 [file MBO3-15-e70304-s002.pptx]

## Slide 1
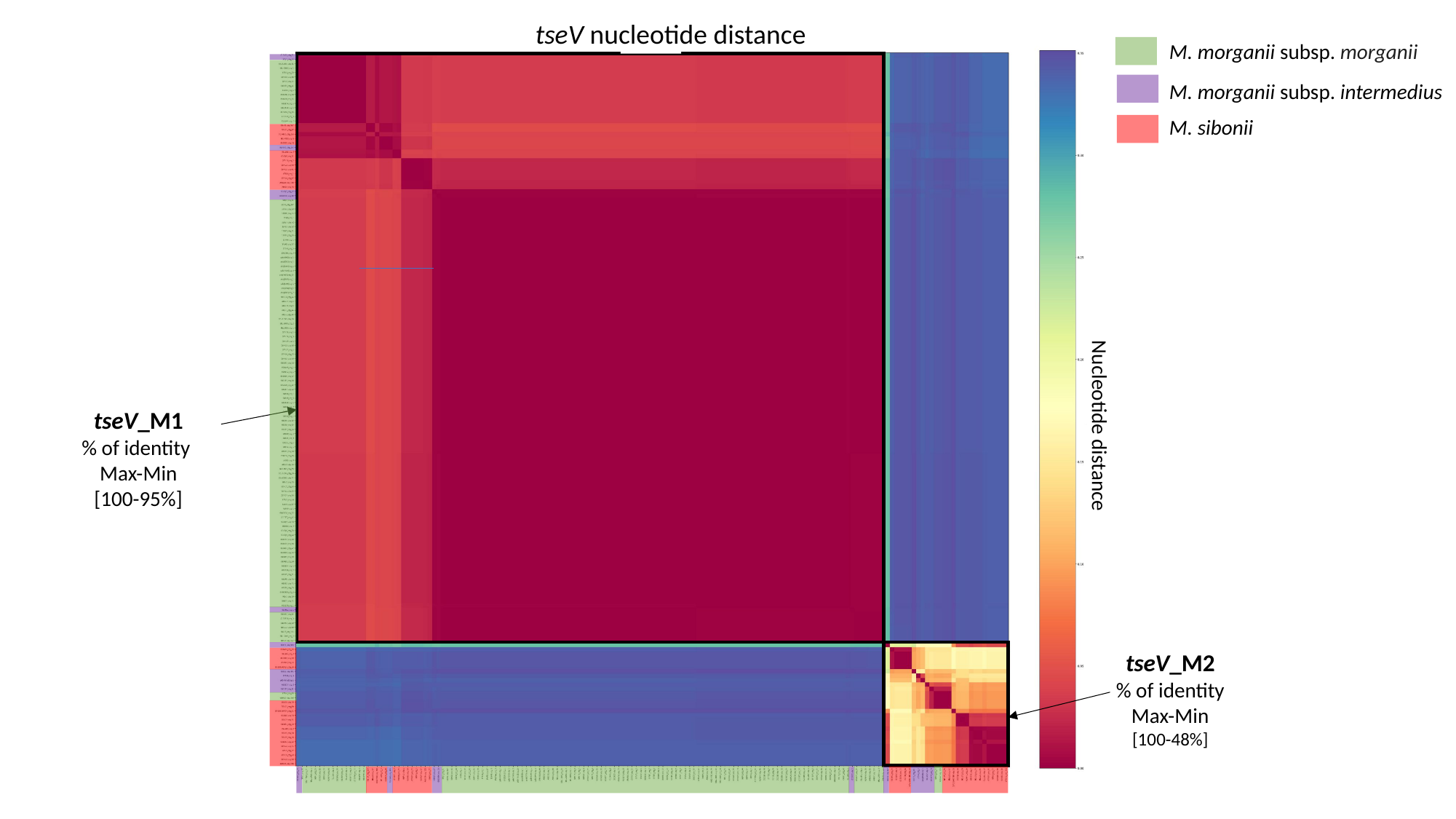

tseV nucleotide distance
M. morganii subsp. morganii
M. morganii subsp. intermedius
M. sibonii
Nucleotide distance
tseV_M1
% of identity Max-Min [100-95%]
tseV_M2
% of identity
Max-Min
[100-48%]
