# Supplementary material for: Genome‐Wide In Silico Analysis of the Type VI Secretion System (T6SS) Within the Morganella Genus
Source: Microbiologyopen. 2026 Apr 30;15(3):e70304. doi: 10.1002/mbo3.70304 (PMC13129497; doi:10.1002/mbo3.70304)
Supplement: Supplementary file 8 — Supporting File 8 [file MBO3-15-e70304-s003.pptx]

## Slide 1
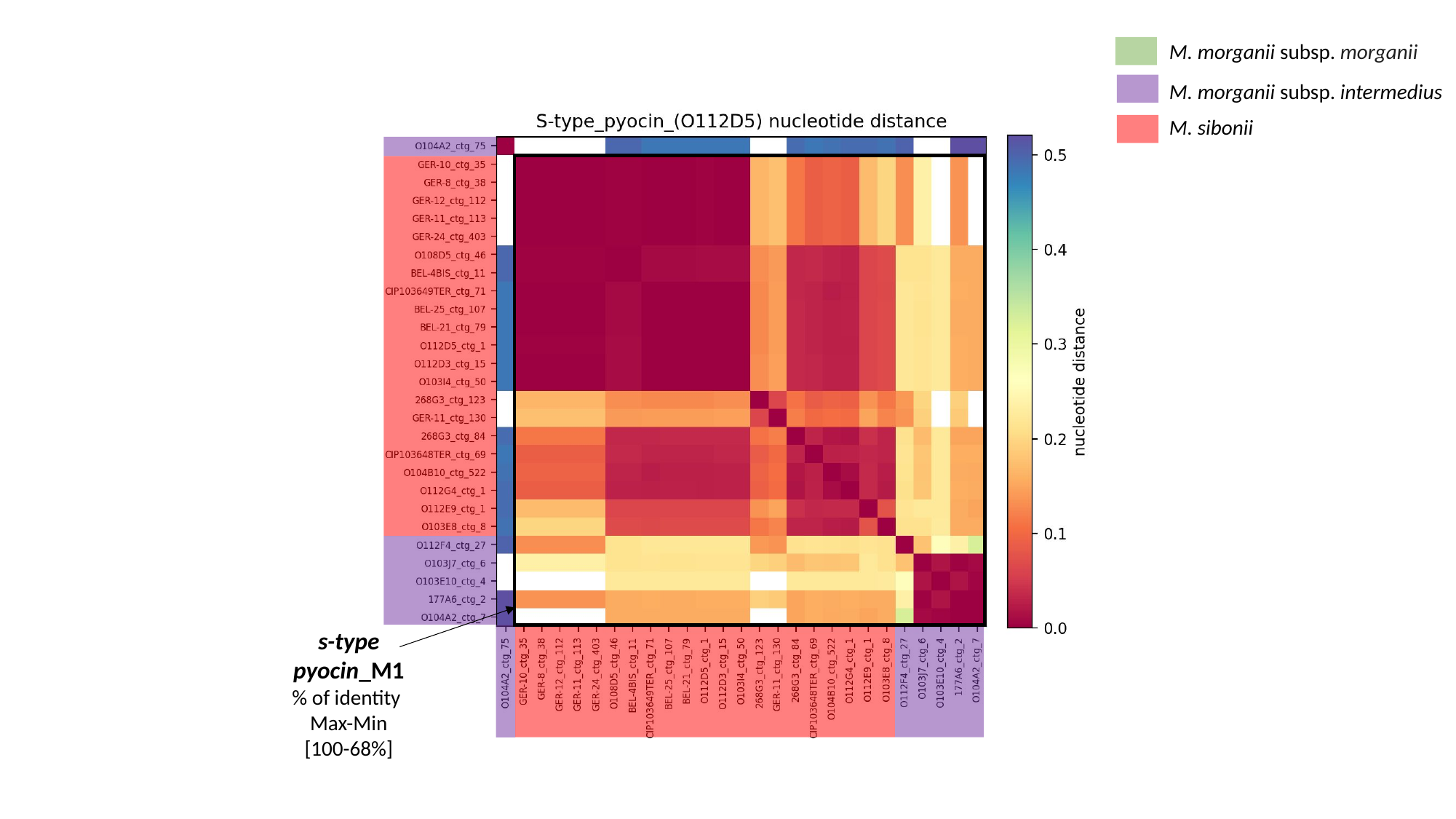

M. morganii subsp. morganii
M. morganii subsp. intermedius
M. sibonii
s-type pyocin nucleotide distance
Nucleotide distance
s-type pyocin_M1
% of identity Max-Min
[100-68%]
